# Biomechanical assessment of two different surgical treatments for the correction of flat foot

**DOI:** 10.1186/1757-1146-7-S1-A52

**Published:** 2014-04-08

**Authors:** Lisa Berti, Giulia Celin, Paolo Caravaggi, Sandro Giannini, Alberto Leardini

**Affiliations:** 1 Movement Analysis Laboratory, Istituto Ortopedico Rizzoli, Bologna, 40136, Italy; 2 1st Division of Orthopedic Surgery, Istituto Ortopedico Rizzoli, Bologna, 40136, Italy

## Introduction

The flat foot is a very frequent deformity in orthopedics and can be observed at different levels of severity already in childhood and infancy. The possible functional alterations associated with flat feet are not fully established. These can result in critical clinical consequences, such as secondary deformities of the forefoot and lower limb, pain and muscle fatigue. The prescription of orthotics or indication for surgical interventions are still much debated. A diagnosis based only on foot morphology is not sufficient to decide the therapeutic approach. In fact, the degree of severity of the deformity and the effects of treatments require also careful functional assessment. This study aims at investigating by means of movement analysis the effects of two different surgical treatments for severe flat foot.

## Methods

Ten children (11.3 ± 1.6 yrs, 19.7 ± 2.8 BMI) were operated for the correction of flat foot [1,2] in both feet. One foot was corrected with a calcaneo-stop method, i.e. a screw implanted into the calcaneus, and the other with an endoprosthesis implanted into the sinus-tarsi. Gait analysis was performed pre- and 12 month post-operative, using a 8-camera motion system (Vicon, UK). An established protocol for lower limb [3] and a multi-segment foot kinematic analysis [4] were used to calculate joint rotations and moments during three walking trials for each subject.

## Results

**Figure 1:**
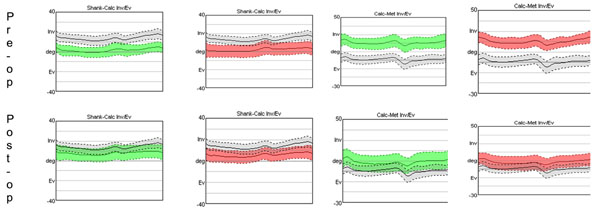
Patterns of joint rotations between calcaneus and shank (two left columns), and between metatarsus and calcaneus (two right columns), during pre-op (top) and at 12 month post-op (bottom). Where in red is the calcaneo-stop group, in green is the endoprosthesis group, and in grey is the control group.

Significant differences in standard X-ray measurements were observed between pre- and post-op, but not between feet. Analysis of the kinematic variables revealed important functional corrections. In particular, joint rotations at the ankle (Figure 1, left) and those between the metatarsus and calcaneus segments (Figure 1, right) improved significantly between pre- and post-op. Ground reaction force showed that the deficits in propulsion and stability pre-op were resolved in both feet, i.e. with both implants.

## Conclusion

The combined lower limb and multi-segment foot kinematics analyses was found adequate and provided a thorough and accurate functional assessment of the entire limbs. Both surgical treatments enabled good restoration of the normal kinematics of the foot and of the lower limb joints. This population will be monitored further to assess the functional progresses in time; preservation, or even improvement, of these results, are expected.

## References

[B1] Giannini S Surgical treatment of flexible flatfoot in children: a four year follow-up study J Bone Joint Surg Am 2001 83-A Suppl 2 Pt 2 73 9 11712838 10.2106/00004623-200100022-00003

[B2] Roth S Minimally invasive calcaneo-stop method for idiopathic, flexible pes planovalgus in children Foot Ankle Int 2007 28 9 991 5 10.3113/FAI.2007.0991 17880873

[B3] Leardini A A new anatomically based protocol for gait analysis Gait Posture 2007 26 4 560 71 10.1016/j.gaitpost.2006.12.018 17291764

[B4] Leardini A Rear-foot, mid-foot and fore-foot motion during the stance phase of gait Gait Posture 2007 25 3 453 62 10.1016/j.gaitpost.2006.05.017 16965916

